# Planning for Serious Illness by the General Public: A Population-Based Survey

**DOI:** 10.5402/2013/483673

**Published:** 2013-12-30

**Authors:** Donna Goodridge, Elizabeth Quinlan, Rosemary Venne, Paulette Hunter, Doug Surtees

**Affiliations:** ^1^College of Nursing, University of Saskatchewan, Saskatoon, SK, Canada S7N 0W8; ^2^Department of Sociology, College of Arts and Science, University of Saskatchewan, 9 Campus Drive, Saskatoon, SK, Canada S7N 5E5; ^3^Edwards School of Business, University of Saskatchewan, 25 Campus Drive, Saskatoon, SK, Canada S7N 5A7; ^4^Department of Psychology, St. Thomas More College, University of Saskatchewan, 1437 College Drive, Saskatoon, SK, Canada S7N 0W6; ^5^College of Law, University of Saskatchewan, 15 Campus Drive, Saskatoon, SK, Canada S7N 5A6

## Abstract

*Background.* While rates of advance care documentation amongst the general public remain low, there is increasing recognition of the value of informal planning to address patient preferences in serious illness. *Objectives.* To determine the associations between personal attributes and formal and informal planning for serious illness across age groups. *Methods.* This population-based, online survey was conducted in Saskatchewan, Canada, in April, 2012, using a nonclinical sample of 827 adults ranging from 18 to 88 years of age and representative of age, sex, and regional distribution of the province. Associations between key predictor variables and planning for serious illness were assessed using binary logistic regression. *Results.* While 16.6% of respondents had completed a written living will or advance care plan, half reported having conversations about their treatment wishes or states of health in which they would find it unacceptable to live. Lawyers were the most frequently cited source of assistance for those who had prepared advance care plans. Personal experiences with funeral planning significantly increased the likelihood of activities designed to plan for serious illness. *Conclusions.* Strategies designed to increase the rate of planning for future serious illness amongst the general public must account for personal readiness.

## 1. Introduction

As community-based practitioners who develop relationships of trust with patients of all ages, family physicians are uniquely situated to incorporate advance care planning into their health promotion initiatives. The benefits of healthy individuals formally expressing their preferences for medical treatment in the event they are unable to communicate their wishes during a serious illness have been widely advocated although uptake of advance care planning by individuals remains highly variable. While research has typically focused on advance care planning in clinical populations for whom death is likely to occur soon (such as those with life-threatening illnesses), less is known about the way in which the general public (including young adults) views and engages in planning for the possibility of future serious illness. An understanding of the factors that may influence patient readiness to engage in planning for serious illness can assist family physicians to sensitively introduce this important topic in their practices.

The primary aim of this population-based survey was to determine the associations between personal attributes and both formal and informal planning activities for future serious illness. Formal planning activities include the preparation of a written advance care plan or the designation of a proxy, while informal activities include conversations with persons close to the individual about states of health which a person would find unacceptable or health care preferences in the event of serious illness. While personal attributes such as sociodemographic characteristics and the presence of health conditions are recognized to influence participation in advance care planning, a novel aspect of our study was exploring whether personal experience with funeral planning is associated with planning for serious illness.

*Background.* Although advance directives have been advocated as a means to provide for a better death for more than two decades, completion rates have remained modest. Advance directive completion rates range between 18% and 36% in the general population amongst American adults [1]. Engagement in the process of advance care planning has been associated with a wide range of factors, including “older age, greater disease burden, type and acuity of condition, White race, higher socioeconomic status, knowledge about advance directives or end of life treatment options, a positive attitude toward end of life discussions, a long-standing relationship with a primary care physician, and whether the patient's primary care physician has a personal advance directive” [1]. Even amongst populations with a greater likelihood of death occurring sooner rather than later (such as older adults and persons with life-limiting illnesses), advance directive completion rates vary widely, ranging from 15% to 84.9% [2–4].

This reluctance of individuals to complete advance directives, in tandem with significant debate about the clinical utility and effectiveness of traditional advance directive documents within the health care community [5–7], has renewed emphasis on *process* in advance care planning. Newer definitions of advance care planning stress the importance of reflection and dialogue, over document completion, in communicating their health care values and preferences as a means to facilitate making the difficult treatment decisions that often occur during a medical crisis.

There is growing evidence that dialogue related to advance care planning is occurring and that the conversations may be achieving positive results [8, 9]. Between 75% and 91% of Canadians over age 65 had thought about the person who would make health decisions for them if they were unable to do so, while 46–69% had discussed their preferences for end of life care with another person [8]. The presence of discussions about end of life care has been found to be associated with less aggressive medical care, including lower rates of ventilation, resuscitation, ICU admission, earlier hospice admission, as well as better patient quality of life and improved bereavement adjustment of families in a group of persons with advanced cancer [9].

In spite of social marketing campaigns designed to heighten awareness of the benefits of advance care planning, myriad reasons for choosing *not* to participate in advance care planning have been described in the literature. These include perceptions of being “too healthy” to require this type of planning; perceptions that advance directives are only for the elderly or infirm and thus irrelevant; difficulties talking about death, including reluctance, denial, anxiety, or sadness associated with discussions of mortality; being “too busy”; not “ready”; lack of knowledge; difficulty of executing the document; reluctance to approach the physician; fear of being a burden; incompatibility with cultural traditions; preference to delegate treatment decision making to family or others; preferring to leave life and death decisions in God's hands; and lack of confidence that an advance care plan will change the treatment received [10–12]. From a societal perspective, denial of the inevitability of death and its “sequestration” from mainstream society [13] have contributed to a “lack of openness” and “taboos” about discussing the end of life and engaging in advance care planning [10].

The Transtheoretical Model [14] provides a model of intentional behavior change which has proven useful as a framework to explain variability in personal “readiness” as a barrier to engagement in advance care planning [5, 15]. According to the model's proponents, people move through stages of precontemplation, contemplation, preparation, action, and maintenance as they consider making a change. In other words, knowing that a change is advantageous (precontemplation) is a number of steps away from actually making the change. A person must also become motivated to make the change (contemplation), plan to make the change (preparation), take action, and maintain that action. For instance, many young couples do not have wills, even though they recognize the potential advantages of having a will. Talking with another couple who has recently made a will may increase motivation, and having a child may further increase that motivation. When motivation is sufficient, the individuals will begin to make plans, such as considering what they want to say in their wills and deciding which lawyer to consult. Ultimately, they will take action to create a will, and, ideally, revisit the terms of the will as needed.

Research using the Transtheoretical Model to explore important behavior changes suggests that significant life events may be “turning points” that catalyze the process of behavior change [16]. On that basis, we hypothesized that personal experiences dealing with death of a close friend or relative may engender new perspectives on dying and death and thus enhance motivation for advance care planning. Relatively few studies have examined personal experiences with the death of a close friend or relative and the willingness to engage in planning for serious illness. In one qualitative study, Lambert and colleagues [17] found that older adults' decisions related to advance care planning were highly influenced by personal experiences with death and by spiritual, emotional, and social considerations. Carr [18] noted that persons whose loved one had prepared an advance directive and died at home with few problems were more likely to engage in advance care planning themselves. Furthermore, 19% of respondents in Carr's [18] study cited others' deaths as a primary motivation for planning. Many of these respondents reported that they wished to avoid experiences they had witnessed in the dying process and had perceived as negative and potentially avoidable, such as pain or use of invasive technology.

Because research exploring the role of life experiences on participation in advance care planning is at a nascent stage, we were particularly interested in exploring whether personal involvement with funeral planning or personal experience with illness might be associated with planning for serious illness amongst the general public.

## 2. Methods

### 2.1. Study Setting, Population, and Design

Given our interest in obtaining a broad cross-section of the general public, we chose to conduct an online, panel-based survey within the province of Saskatchewan, Canada, in April, 2012. Eight hundred and twenty-seven residents aged 18 years and older were randomly selected from a large pool of individuals who had contracted to participate in commercial market surveys (SaskWatch Research). Participants were stratified by age, sex, and region to be representative of the demographic characteristics of the province. Ethical approval was obtained from the University of Saskatchewan Behavioral Review Board. Participants consented to the data they provided being used in subsequent presentations and publications.

### 2.2. Description of Variables

The survey was comprised of structured, closed-ended items salient to planning for serious illness care. Demographic data included age, sex, personal income, education, and residence (urban or rural). Respondents were asked to identify the number and type of health conditions with which they lived from a list of common illnesses, as well as to rate the importance of planning for medical care at the end of life and the number of times they had been involved in funeral planning for a relative or close friend (never, once, or two or more times). Outcome measures included rating of the importance of planning for medical care in the event the individual could not communicate their wishes for treatment; frequency of discussions with persons close to the respondent about wishes for treatment in case of serious illness; frequency of discussions with persons close to the respondent about health states in which the respondent would find unacceptable to live; and completion of a written advance care plan. Persons who reported a written living will or advance directive were also asked to identify sources of help received with preparing this document.

### 2.3. Data Analysis

Statistical analysis was completed using SPSS 19.0. Level of significance (*σ*) was set at 0.05. Comparisons between the groups were completed using the Kruskal-Wallis test (with multiple Mann-Whitney tests adjusted with Bonferroni corrections for post hoc analysis) and chi-square tests of proportion where appropriate. Associations between key predictor variables and with planning for serious illness were assessed using binary logistic regression. The strength of association was measured by the odds ratio (OR) and 95% confidence intervals (CI).

## 3. Results

Table 1 displays the sociodemographic characteristics of the sample by age group and overall. Respondents ranged in age from 18 to 88 years. The participation rate was 100%. The sample generally reflected the age, sex, and regional distribution of the province. For example, 13.5% of participants to the survey were aged 65 and older, compared with the census-derived proportion of 14.9% for this age group living in Saskatchewan [19]. Forty percent of participants reported never having been involved in planning a funeral for a relative or close friend, while 36% had been involved two or more times.

Table 2 displays the bivariate associations between the demographic, health and experiential variables and beliefs, and behaviors related to planning for serious illness. The majority (76.3%) of respondents indicated they felt it was important or very important to plan for medical care in the event of serious illness. The proportion who expressed this belief was significantly higher among women, those who had completed postsecondary education and persons who had been involved in planning one or more funerals. Just over half (51.4%) of the sample reported having conversations about their treatment wishes, and 46.6% had engaged in conversations regarding states of health in which they would find it unacceptable to live.

Discussions relating to preferences for treatment and unacceptable conditions were associated with the number of health conditions and having been involved with planning one or more funerals. The majority (83.4%) of respondents did not have a written advance care plan although the proportions of those who did increase in a stepwise fashion with advancing age. For the 137 respondents who reported having a written advance care plan, lawyers were most frequently (47.4%) cited as a source of assistance in preparing this document. Five per cent of those with advance care plans indicated they had consulted a physician in preparing their advance care plan. A substantial proportion (21.2%) of the advance care plans were prepared solely by the individual with no consultation from other sources.

After adjustment for all the variables listed in Table 3, having been involved in planning one or more funerals independently increased the likelihood of all outcomes related to planning for serious illness. Persons aged 55–64 years were less likely than either younger or older individuals to have had a discussion in the past year about health states in which they would find it unacceptable to live. Women were twice as likely as men to rate planning as important, while persons with two or more health conditions were more likely than the reference group to have discussed wishes for medical treatment as well as unacceptable conditions. Persons aged 65 and older were much more likely than younger age groups to have completed an advance care plan, while persons living in rural areas were less likely to have such a document compared to those in larger urban centres.

Our findings demonstrate that personal experience in dealing with the death of people close to an individual, such as involvement in funeral planning, is independently associated with key attitudes and behaviors related to planning for serious illness and a better predictor than the presence of multiple medical conditions. Previous involvement in funeral planning was the single predictor variable independently associated with all outcomes studied (importance of planning for care in the event of serious illness; having had a discussion with those close to the individual about wishes for medical treatment in the event of incapacity; having had a discussion with those close to the individual about health states that would be unacceptable to live with; and completion of a living will or advance care plan). Involvement with funeral planning may represent a “turning point” that promotes receptivity to advance planning activities for bereaved individuals and a potential opportunity for family physicians to initiate these important discussions.

The relative absence of physician involvement in preparing advance care plans was somewhat surprising, although not entirely unexpected. Physicians were rarely cited as a source of assistance for respondents who had prepared written living wills or advance care plans in our study, providing further corroboration for similar results from Teno and colleagues [20]. While organizational advocates of advance care planning frequently include general practitioners as a key resource for patients [21, 22], there has been little research explicating the way in which practitioners of family medicine enact this role [23]. Gallagher [24] suggests initiating discussions about advance care planning in primary care using the following three questions: (a) What present or future experiences are most important for you to live well at this time in your life?; (b) What fears or worries do you have about your illness of medical care?; and (c) What sustains you when you face serious challenges in life? [25].

Members of the public rely heavily on lawyers to prepare planning documents. Close to half of respondents who had completed a written advance care plan did so in consultation with a lawyer, reinforcing that legal professionals play a key role in planning for serious illness amongst the general public. Lawyers' professional training focuses on the legal aspects of end of life planning. Whether drafting estate wills or advance care plans, lawyers are trained to understand the default legislative provisions in their jurisdiction, the flexibility individuals have to alter the default regime, and the legal procedure necessary to bring about this alteration. This method of giving voice to the clients' wishes may cause many lawyers to view end of life decisions through a legal rather than a holistic lens, potentially limiting the clinical utility of advance care plans, as previously discussed. New forms of interprofessional collaborations should be considered to increase the interface between physicians and lawyers. Advance care planning clinics in which lawyers work alongside health care providers could provide a value-added services to clients interested in preparing formal documents.

There is dialogue occurring amongst members of the public about planning for future serious illness. While physicians may be reluctant to raise this issue with their patients, approximately half of the respondents indicated that they had had discussions with someone close about either their wishes for medical care in the event of incapacity or about health states in which they would find it unacceptable to live. The relatively high proportion (69.6%) of respondents in the oldest age category who had discussed their wishes for medical care with a person close to them is consistent with the results of the Canadian Study on Health and Aging [8], in which 46–69% of older persons reported having had such a conversation.

Age *per se* was not demonstrated to necessarily promote planning for serious illness. After adjusting for all included variables, older age was associated only with greater likelihood of completing an advance care plan, possibly the result of discussions with lawyers about more general estate planning.

Persons aged 55 and older were much more likely to be living with one or more health conditions, yet were much less likely than persons aged 18–34 to have engaged in discussions about health states in which they would find it unacceptable to live. Generational differences in communication patterns may contribute to older adults' greater reluctance to engage in discussions about unacceptable health states, compared to young adults who claim to have little problem debating such issues [21, 22]. Gender was associated with beliefs about planning for serious illness, which was not surprisingly given women's well-known roles as caregivers, greater life expectancies, and higher likelihood of widowhood.

While our survey was representative of the population of the province in terms of age, sex, and region, we recognize the limitations of this study. The sample was randomly selected from only those individuals who had agreed to participate in online surveys conducted by SaskWatch Research and not from the entire population of the province. The sample was not representative of other factors, such as income, ethnicity or religious affiliation that may influence planning for serious illness. The survey nature of the design precluded in-depth exploration of the range of factors important to advance care planning. The analysis is based on self-reports. The cross-sectional survey design provides a snapshot of a single point in time, rather than allowing us to establish any type of causal link between involvement in funeral planning and planning for serious illness.

## 4. Conclusions

While planning for serious illness is valued by the majority of the general public, there continues to be limited uptake of formal planning activities such as written advance care plans. There is evidence, however, that patients may be taking part in informal planning, such as discussions about important values such as treatment wishes and health states that would be considered unacceptable to live in. Given their ongoing relationships with patients, family physicians are uniquely situated to provide knowledgeable and reasoned support to individuals who express interest in planning for serious illness although the role of family physicians in advance care planning may be fairly limited at present. For some patients, involvement in funeral planning may increase awareness of their own vulnerability and heighten interest and readiness to engage in discussions about planning for serious illness with their family physicians. Together with their patients, family physicians have a significant role to play ensuring that patients are able to effectively plan for future care that is appropriate to and respectful of personal values and preferences.

## Figures and Tables

**Table 1:** Sociodemographic, health, and experiential characteristics of the sample (by age group and overall).

	18–34 years *n* = 237 %	35–54 years *n* = 307 %	55–64 years *n* = 171 %	>65 years *n* = 112 %	Overall *n* = 827 %
Sex					
Male	46.0	49.2	45.6	47.3	47.3
Female	54.0	50.8	55.4	52.7	52.7
Education					
< or completed high School	12.4	17.8	20.1	14.4	16.1
Some postsecondary	56.0	54.3	52.1	55.0	53.8
completed Postsecondary	31.6	28.0	27.8	30.6	29.0
Income					
<$30,000	14.3	6.8	7.6	11.6	9.8
$30,000 to $59,999	27.4	16.6	15.8	30.4	21.4
$60,000–89,999	19.0	18.9	14.6	19.6	18.1
<$90,000	21.3	38.4	35.1	8.9	28.9
Refused	23.3	19.2	26.9	29.5	21.8
Residence					
Large urban	49.4	34.2	43.3	41.1	41.4
Other	50.6	65.8	56.7	58.9	58.6
Health conditions					
None	56.5	44.3	27.5	10.7	39.8
One	28.7	28.3	29.2	19.6	27.4
Two or more	14.8	27.4	43.3	69.6	32.8
Involved in funeral planning for relative or close friend					
Never	74.3%	36.5%	17.5%	9.8%	39.8%
Once	19.8%	29.3%	24.6%	18.8%	24.2%
Twice or more	5.9%	34.2%	57.9%	71.4%	36.0%

**Table 2:** Variables associated with serious illness planning beliefs and behaviors.

	Important or very important to plan for care	Have discussed wishes for treatment	Have discussed unacceptable states of health	Prepared written advance care plan or living will
Overall	76.3%	51.4%	46.6%	16.6%
Sex				
Male	69.0%^¶^	47.1%^†^	46.3%	15.3%
Female	82.8%^¶^	55.3%^†^	46.8%	17.7%
Age				
18–34	75.9%	43.5%	40.9%	8.9%^†^
35–54	74.6%	49.5%	47.2%	14.3%^†^
55–64	76.6%	53.8%	46.8%	20.5%^†^
≥65	81.3%	69.6%	56.3%	33.0%^†^
Education				
< or completed High school	69.2%	48.9%	41.4%^†^	17.3%
Some postsecondary	77.1%	53.0%	50.1%^†^	17.3%
Completed Postsecondary	79.2%	49.2%	42.5%^†^	15.4%
Income				
<$30,000	75.3%	53.1%	44.4%	18.5%
$30,000 to $59,999	74.6%	54.2%	47.5%	16.4%
$60,000–89,999	78.0%	46.0%	44.7%	12.5%
<$90,000	75.7%	51.0%	47.3%	17.6%
Refused	77.8%	52.9%	47.2%	17.8%
Residence				
Large urban	74.9%	52.3%	47.7%	19.0%
Other	77.3%	50.7%	45.8%	14.8%
Health conditions				
None	73.3%	43.2%^†^	39.2%^¶^	12.2%
One	75.3%	50.2%^†^	44.9%^¶^	18.1%
Two or more	80.8%	62.4%^†^	56.8%^¶^	20.7%
Involved in funeral planning				
Never	70.2%*	36.5%^¶^	34.3%^¶^	7.9%^¶^
Once	80.0%*	52.5%^¶^	44.0%^¶^	13.5%^¶^
Twice or more	80.2%*	67.1%^¶^	61.7%^¶^	28.8%^¶^

*P < 0.10.

^†^ *P* < 0.05.

^¶^ *P* < 0.001.

**Table 3:** Adjusted^1 ^associations between respondent characteristics and aspects of planning for serious illness.

	Important or very important to plan for care	Discussed treatment wishes OR (95% CI)	Discussed unacceptable conditions OR (95% CI)	LW or ACP completed OR (95% CI)
Female	2.05^¶^	0.75	1.04	0.91
(ref: male)	(1.45–2.90)	(0.56–1.01)	(0.77–1.40)	(0.61–1.36)
Age 35–54 years	0.69	0.87	0.87	1.17
(ref: <18–34 years)	(0.44–1.07)	(0.59–1.29)	(0.59–1.28)	(0.63–2.16)
Age 55–64 years	0.67	0.75	0.58^†^	1.33
(ref: <18–34 years)	(0.38–1.16)	(0.46–1.20)	(0.29–0.78)	(0.67–2.60)
Age ≥65 years	0.76	1.20	0.65^†^	2.47^‡^
(ref: <18–34 years)	(0.52–1.84)	(0.67–2.14)	(0.37–1.14)	(1.19–5.14)
Some postsecondary education	1.51	1.25	1.51^†^	1.04
(ref: ≤high school)	(0.96–2.36)	(0.83–1.90)	(1.01–2.28)	(0.60–1.78)
Completed postsecondary	1.82^†^	1.07	1.08	0.86
(ref: <high school)	(1.08–3.05)	(0.68–1.71)	(0.68–1.72)	(0.46–1.61)
Income of $30,000–59,999	0.97	1.04	1.04	0.81
(ref: <$30,000)	(0.51–1.83)	(0.59–1.82)	(0.59–1.83)	(0.39–1.70)
Income of >$60,000–89,999	1.21	0.75	0.91	0.61
(ref: <$30,000)	(0.62–2.37)	(0.42–1.34)	(0.52–1.83)	(0.28–1.36)
Income ≥$90,000	1.04	0.93	0.91	0.90
(ref: <$30,000)	(0.54–2.01)	(0.53–1.64)	(0.52–1.59)	(0.43–1.89)
Income unspecified	1.15	0.74	0.84	0.73
(ref: <$30,000)	(0.82–1.63)	(0.42–1.31)	(0.62–1.13)	(0.35–1.51)
Rural residence	1.15	0.86	0.85	0.67*
(ref: urban)	(0.82–1.63)	(0.64–1.20)	(0.62–1.16)	(0.45–0.99)
One health condition	0.96	1.09	1.17	1.23
(ref: 0 conditions)	(0.64–1.44)	(0.76–1.56)	(0.82–1.69)	(0.75–2.03)
Two or more health conditions	1.33	1.53^†^	1.76^‡^	1.02
(ref: 0 conditions)	(0.86–2.07)	(1.05–2.21)	(1.22–2.56)	(0.61–1.68)
Involved in planning one funeral	1.73^†^	1.89^‡^	1.59^†^	1.62
(ref: no previous involvement in funeral)	(1.10–2.72)	(1.05–2.34)	(1.07–2.34)	(0.88–2.97)
Involved in planning two or more funerals	1.83^†^	3.38^¶^	3.48^¶^	3.54^¶^
(ref: no previous involvement in funeral)	(1.16–2.88)	(2.26–5.06)	(2.32–5.21)	(2.00–6.27)

^1^Adjusted for each of the variables listed in the table.

*P < 0.10.

^†^ *P* < 0.05.

^‡^ *P* < 0.01.

^¶^ *P* < 0.001.
